# Severe Usutu Virus Encephalitis Treated With Corticosteroids: A Case Report

**DOI:** 10.1155/crdi/2284329

**Published:** 2026-07-24

**Authors:** Stefania Casolari, Deborah Lusetti, Elena Martini, Licia Gozzi, Gabriella Orlando, Erica Franceschini, Cristina Mussini

**Affiliations:** ^1^ Infectious Diseases Unit, Azienda Ospedaliero-Universitaria di Modena, University of Modena and Reggio Emilia, Modena, Italy, unimore.it; ^2^ Department of Surgical, Medical, Dental and Morphological Sciences, University of Modena and Reggio Emilia, Modena, Italy, unimore.it

**Keywords:** flavivirus neuroinvasive disease, steroid therapy, Usutu virus, viral encephalitis

## Abstract

Usutu virus (USUV) is a neurotropic flavivirus, phylogenetically related to West Nile virus (WNV), maintained in the environment through a bird‐to‐mosquito transmission cycle, with humans and other animals occasionally becoming infected. In recent years, different cases of human infections have been reported concomitantly with increased viral circulation in several European countries. Most USUV infections in humans remain asymptomatic; however, neuroinvasive disease has been reported, mainly in the elderly and the immunocompromised but also in healthy individuals. Herein, we report a case of severe USUV meningoencephalitis in an elderly patient treated with a short course of dexamethasone and discuss the potential role of steroids in light of the most recent literature on the pathogenetic mechanisms of arboviral encephalitis.

## 1. Introduction

Usutu virus (USUV) is an arthropod‐borne virus that has recently gained attention for its ability to cause infections of the central nervous system. It belongs to the Japanese encephalitis virus (JEV) serocomplex and is a member of the Orthoflavivirus genus, within the Flaviviridae family [1]. It is an enveloped, single‐stranded RNA virus whose natural transmission cycle involves mosquitoes as vectors (mainly *Culex* spp. but also the *Aedes* and *Mansonia* genera), birds as amplifying hosts, and humans and other mammals as incidental “dead‐end” hosts [2]⁠. As transmission requires competent vectors, in temperate climates, virus circulation is limited to some periods of the year, usually from late spring to early autumn.

Most human USUV infections are asymptomatic or present with mild and nonspecific symptoms (i.e., rash, fever, and headache). However, neurological disorders ranging from facial paralysis to severe forms of meningoencephalitis and encephalitis have also been reported [3]⁠. Laboratory diagnosis of USUV infection is classically based on the detection of the USUV RNA genome by reverse transcription polymerase chain reaction (RT‐PCR) in bodily fluids or on the demonstration of a specific antibody response against the virus. However, serological tests for USUV suffer from a lack of specificity and must be controlled by neutralization approaches to exclude cross‐reaction with antibodies directed against other flaviviruses, including West Nile virus (WNV) [4, 5]. USUV can also be isolated in cell culture, but this method is not routinely indicated for diagnosis due to its complexity, costs, and long turnaround times.

First isolated in South Africa in 1959, USUV is thought to have been introduced into Europe through bird migration [6]. The first human case of USUV infection was reported in 1981 in the Central African Republic, and a second case was identified in Burkina Faso in 2004; both patients experienced moderate symptoms including fever, rash, and jaundice [7]. In 2009, the first case of USUV encephalitis was diagnosed in Modena, in the north of Italy, in a patient with B‐cell lymphoma. In the same year, a second case of neuroinvasive infection was reported in a patient recovering from orthotopic liver transplantation [8, 9].

Over the last two decades, USUV has spread rapidly across the continent, becoming endemic in several countries, with a rise in reported human infections [3, 10, 11]. Seroprevalence studies conducted in Europe suggest that human USUV infections are largely underestimated. Antibodies against USUV were detected in the blood of 0.01% and 1% of healthy blood donors in Germany and Italy, respectively [12, 13], while a higher seroprevalence (7.5%) was reported in serum samples collected from healthy individuals in Serbia [14]. A retrospective analysis conducted in Italy on 915 patients, about one‐third of whom were suspected of having encephalitis or meningoencephalitis, found a seroprevalence of 6.5% and USUV RNA in 1.1% of cerebrospinal fluid (CSF) and serum samples. Moreover, the study detected an intense cocirculation with WNV in that area, with significantly higher USUV RNA presence and USUV antibody levels compared to those of WNV [15]⁠.

Since 2017, Italy has implemented a joint USUV and WNV national surveillance plan, integrated within a One Health approach, targeting humans, birds, equids, and mosquitoes, with the goal to identify areas at high risk of human disease. Indeed, in Italy, USUV infection is a notifiable disease [16]⁠.

In 2023, the ECDC published the first surveillance report of WNV and USUV infections in the EU/EEA, providing an overview of the epidemiological situation. Eight countries (Austria, Croatia, Czechia, France, Germany, Hungary, Italy, and the Netherlands) reported a total of 104 cases of autochthonous human USUV infections between 2012 and 2021, of which 56 were found in Italy (54%) [11]⁠.

As far as we know, more than 30 cases of acute human infection with neurological symptoms have been described, most in patients with comorbidities of varying severity, although some developed neurological forms without any identified concomitant pathological conditions [3]⁠. To date, there is no approved antiviral drug for USUV and for other arboviral infections, and treatment remains supportive and symptomatic. While there are some conflicting case reports on the use of steroids in viral encephalitis (VE), overall, the data are unfavorable, demonstrating no benefit to support their use [17–20].

There is also concern that steroids might lead to uncontrolled viral replication and potential adverse events.

We present a case of severe USUV encephalitis in which we attempted to leverage the anti‐inflammatory properties of corticosteroids.

## 2. Case Report

In August 2024, an 84‐year‐old man presented to the Emergency Department in Modena Hospital (Italy) with a 1‐week history of fever and chills. His past medical history included only hypertension and dyslipidemia. The patient was independent in activity of daily living (ADL) and instrumental activity of daily living (IADL).

Upon admission, despite high fever (39°C), the patient was alert, oriented to person, and cooperative, with a Glasgow Coma Scale score of 15. Vital signs revealed a blood pressure of 115/65 mmHg and a heart rate of 105 beats per minute (bpm). Arterial blood gas analysis documented respiratory failure (SpO2 84%). Blood tests showed leukopenia (WBC 2600/mmc, N 1550/mmc), anemia (Hb 10.9 g/dL), mild renal impairment (creatinine 1.25 mg/dL; eGFR 53 mL/min), and C‐reactive protein 1.5 mg/dL. A chest X‐ray revealed bilateral lower interstitial thickening. A molecular swab for SARS‐CoV‐2 was negative.

After admission to the geriatrics department, blood and urine cultures were collected, and treatment with ceftriaxone was started. Over the following 24 h, fever persisted, and the patient’s mental status deteriorated, with the onset of confusion, drowsiness, aphasia, buccal synkinesis, distal tremors, and neck stiffness. Plain brain computed tomography and electroencephalogram were both unremarkable. Lumbar puncture revealed slightly cloudy CSF, with pleocytosis (122 cells/μL, mixed mononuclear and polymorphonuclear), elevated protein (137 mg/dL), and mild hypoglycorrhachia (54 mg/dL, with peripheral blood glucose 120 mg/dL).

Since two doses of ceftriaxone had already been administered before the lumbar puncture, and given the presence of neutrophils in the CSF, empirical treatment with ceftriaxone, ampicillin, and acyclovir was started despite viral infection being suspected. Due to clinical severity, i.v. dexamethasone 12 mg was also administered. The patient was transferred to a subintensive care unit. A qualitative multiplex polymerase chain reaction (PCR) for common meningitis/encephalitis pathogens (BioFire FilmArray meningitis/encephalitis panel) on CSF gave a negative result, as well as later cultures.

CSF, serum, and urine samples were sent to the Regional Reference Centre for Microbiological Emergencies (CRREM) to be tested for arboviral infection. The following day, tests gave positive results for USUV by RT‐PCR [21] in serum and urine, while results for CSF were negative. Serological and molecular assays for WNV and TOSV were negative. No serology for USUV was performed. All antibiotics and antiviral treatments were subsequently discontinued.

Given that rapid neurological improvement in consciousness and resolution of neck stiffness occurred, we decided to administer a second dose of dexamethasone (reduced to 8 mg) the day after the first infusion. A few hours later, the patient began to speak again and achieved full restoration of consciousness; steroid administration was then discontinued. In the following days, gradual improvement of asthenia, buccal synkinesis, and tremors was observed. At discharge, the patient was in good general condition, independent in ADL, partially dependent in IADL (6/8), and able to walk independently with a walking stick.

## 3. Discussion

Currently, USUV is endemic in Europe, and its spread to new geographic areas is expected to rise, together with the potential occurrence of severe forms of encephalitis [22]. VE is a major cause of morbidity and mortality worldwide; however, with the exception of acyclovir for Herpes simplex virus (HSV) encephalitis, there is no treatment supported by high‐quality evidence from clinical trials. Immunomodulatory therapy with glucocorticoids has been widely used, either as an adjunct to antiviral drugs or as monotherapy, when no effective agent is available.

Although evidence supporting the routine use of steroids remains scant, they are often administered based on individual clinical judgment or local policy, adopting a personal approach regarding dosage, duration of treatment, and timing of initiation. The rationale behind corticosteroid administration lies in their potential to reduce the exaggerated inflammatory response that characterizes the pathogenic mechanisms of VE. Indeed, the neurological damage in VE seems to be due to an uncontrolled immune system reaction of the host—a cytokine storm—rather than a direct viral invasion [17].

After a neurotropic virus infects the brain, an immune cascade leads to cytokine release, recruiting leukocytes to infiltrate the perivascular space and parenchyma. While this response is essential for viral clearance, overexpression of inflammatory cytokine genes may be detrimental in the long term to neuronal function, enhancing the severity of the inflammation. Results from different studies have confirmed that high levels of proinflammatory cytokines adversely affect the blood–brain barrier (BBB) integrity, facilitating viral entry into the brain and vascular leakage, leading to brain edema and increased intracranial pressure [23–26].

On the other hand, there are concerns that immunosuppressive effects could exacerbate viral replication, particularly during early infection, resulting in worsening of the disease. Although never demonstrated, this has led many practitioners to restrict corticosteroid use to patients with significant brain edema.

According to the 2008 IDSA guidelines for VE, there is poor‐quality evidence to support a recommendation for glucocorticoid use in patients with encephalitis due to HSV, EBV, or VZV [27]. A clinical trial investigating adjunctive steroids in HSV encephalitis (GACHE trial) was terminated prematurely due to slow recruitment, after enrollment of only 21 patients in the steroid arm and 20 patients in the placebo arm. No significant differences were seen in primary or secondary endpoints, and no serious adverse effects occurred in patients treated with dexamethasone [28, 29]. Hodzic et al., in a recent meta‐analysis, concluded that steroid use in VE cannot be routinely recommended for lack of benefit, underscoring the poor quality of data, limited by heterogeneity of study populations and interventions, incomplete data, and different bias [17]. The newly released DexEnceph trial, the only completed RCT investigating the effects of adjunctive dexamethasone in HSV encephalitis, did not find any improvement in the primary outcome (verbal memory) and no significant differences in secondary outcomes, including other neuropsychological, functional, or imaging outcomes [19, 20]⁠. The authors concluded that their findings cannot support the adjunction of steroids in HSV encephalitis.

Concerning flavivirus encephalitis, corticosteroid use for WNV encephalitis has been proposed in case reports and clinical studies, with conflicting results [30, 31]. Real‐life data from a retrospective multicentric Italian study found no significant impact on intrahospital mortality or neurological sequelae, highlighting the need for prospective studies [18]⁠. A recent systematic review of WNV encephalitis pathogenesis and related inflammation identifies IL‐6 and TNF‐α as the principal cytokines upregulated during CNS infection. They both exert protective effects during infection, but they are also capable of inducing inflammation‐related damage, resulting in effects that are detrimental to the host [32]. Immunomodulation therapy could mitigate the cytokine burden of inflammation.

Patabendige et al. conducted an elegant in vitro study, using a BBB model inoculated with JEV. The authors observed that JEV infection triggered cytokine production from brain endothelial cells and astrocytes, controlling viral replication but disrupting BBB integrity, allowing entry of the virus into the brain. Dexamethasone administration dampened the immune response and restored barrier integrity without increasing JEV particles in culture, supporting reconsideration of its therapeutic role [33]⁠. A recent research using a mouse model of USUV encephalitis showed that microglial activation, BBB disruption, and inflammatory leukocyte recruitment are hallmarks of disease. Dexamethasone treatment prevented proinflammatory mediator production and reduced leukocyte recruitment significantly, restraining encephalitis severity in mice. Considering that extensive inflammation, rather than the viral load in the brain, is at the root of USUV‐induced disease burden, the authors suggest that steroids could have a role in the treatment of USUV encephalitis [34]⁠.

With regard to safety, it is known that even a short course of corticosteroids can cause side effects such as acute psychosis, exacerbation of diabetes, thrombotic events, and worsening of systemic infections. However, even if available studies do not demonstrate a clear impact of steroids on mortality or neurological outcomes, they consistently highlight a negligible risk of adverse events. The previously cited meta‐analysis by Hodzic et al. found no safety concerns, particularly regarding survival [17]. Similarly, in an old trial by Hoke et al. examining the effects of high‐dose corticosteroids in JEV patients, no harmful effects of treatment could be demonstrated [35]. An observational cohort study conducted in Denmark also found no dose‐dependent association between dexamethasone and unfavorable outcomes in patients with viral meningitis [36]⁠. In the DexEnceph RCT, administration of dexamethasone 40 mg daily for 4 days was not associated with harm and importantly, and it did not lead to an increased detection of HSV in the CSF at 14 days. Indeed, the authors concluded that although it was not possible to demonstrate an improvement in patient outcome, considering that steroids have a role in the treatment of inflammatory brain disease such as autoimmune encephalitis, the safety in HSV encephalitis raises the possibility for early empirical therapy in suspected encephalitis, even before the cause is established [19, 20].

## 4. Conclusion

Due to the paucity of reported cases, USUV‐induced encephalitis remains an almost unexplored field. We consider this clinical case of particular interest, as it raises several important considerations.

First, from an epidemiological point of view, many areas in Europe are characterized by the cocirculation of USUV and WNV. Given the cross‐reactivity of the diagnostic tests between these viruses, USUV neuroinvasive disease may be underreported, as some USUV infections could be misclassified as WNV infections.

Second, USUV encephalitis may evolve quickly: It is important to maintain a high level of suspicion in endemic areas, in order to avoid diagnostic delays, especially in the elderly and the immunocompromised patients.

Third, in light of the literature hypothesizing a central role of the immune response in the pathogenetic mechanism of arboviral encephalitis and considering that available current research aligned in identifying a low risk of harmful effects in this context, we believed that an early short‐term immunomodulatory treatment with dexamethasone could be beneficial. The prompt clinical and neurological improvement observed after only two doses of dexamethasone was impressive.

The dose we selected was well below the 40 mg recommended in bacterial meningitis and consistent with studies reporting favorable outcomes, where most patients received dexamethasone at equivalent doses of 5–30 mg per day [17].

At the time we treated our patient, results from DexEnceph were not available yet. As this is the only RCT on VE and steroids, it can be worth citing one reported study limitation: Although the primary outcome showed no difference, the 95% CI (−9.6–13.1) includes potential for clinically meaningful effects, therefore a clinically relevant benefit cannot be excluded [19, 20].

We hope that this case, together with the most recent literature on the pathogenetic mechanism of arboviral encephalitis, may encourage further investigation into the potential role of corticosteroids as a treatment option for USUV and other closely related flavivirus‐associated neurological disorders, at least until more targeted immunomodulatory therapies will be available.

**Nomenclature**
ADL: Activity of daily living
BBB: Blood–brain barrier
CSF: Cerebrospinal fluid
EBV: Epstein–Barr virus
HSV: Herpes simplex virus
IADL: Instrumental activity of daily living
JEV: Japanese encephalitis virus
TOSV: Toscana virus
USUV: Usutu virus
VE: Viral encephalitis
VZV: Varicella zoster virus
WBCs: White blood cells
WNV: West Nile virus

## Funding

No funding was obtained.

## Disclosure

All authors read and approved the final manuscript.

## Ethics Statement

The authors have nothing to report.

## Consent

We obtained informed and signed consent for publication from the patient.

## Conflicts of Interest

The authors declare no conflicts of interest.

## Data Availability

Data sharing is not applicable to this article as no datasets were generated or analyzed during the current study.
